# Intraclade Heterogeneity in Nitrogen Utilization by Marine Prokaryotes Revealed Using Stable Isotope Probing Coupled with Tag Sequencing (Tag-SIP)

**DOI:** 10.3389/fmicb.2016.01932

**Published:** 2016-12-02

**Authors:** Michael Morando, Douglas G. Capone

**Affiliations:** Marine and Environmental Biology, University of Southern CaliforniaLos Angeles, CA, USA

**Keywords:** microbial diversity, functional heterogeneity, stable isotope probing, nitrogen cycling, new production, microbial subpopulations, nitrate assimilation, ecotype

## Abstract

Nitrogen can greatly influence the structure and productivity of microbial communities through its relative availability and form. However, the roles of specific organisms in the uptake of different nitrogen species remain poorly characterized. Most studies seeking to identify agents of assimilation have been correlative, indirectly linking activity measurements (e.g., nitrate uptake) with the presence or absence of biological markers, particularly functional genes and their transcripts. Evidence is accumulating of previously underappreciated functional diversity in major microbial subpopulations, which may confer physiological advantages under certain environmental conditions leading to ecotype divergence. This microdiversity further complicates our view of genetic variation in environmental samples requiring the development of more targeted approaches. Here, next-generation tag sequencing was successfully coupled with stable isotope probing (Tag-SIP) to assess the ability of individual phylotypes to assimilate a specific N source. Our results provide the first direct evidence of nitrate utilization by organisms thought to lack the genes required for this process including the heterotrophic clades SAR11 and the Archaeal Marine Group II. Alternatively, this may suggest the existence of tightly coupled metabolisms with primary assimilators, e.g., symbiosis, or the rapid and efficient scavenging of recently released products by highly active individuals. These results may be connected with global dominance often seen with these clades, likely conferring an advantage over other clades unable to access these resources. We also provide new direct evidence of *in situ* nitrate utilization by the cyanobacterium *Prochlorococcus* in support of recent findings. Furthermore, these results revealed widespread functional heterogeneity, i.e., different levels of nitrogen assimilation within clades, likely reflecting niche partitioning by ecotypes.

## Introduction

The nitrogen (N) cycle is complex and N availability can regulate primary production and, in turn, determine where and how new biomass is formed, stored, and exported from the euphotic zone of aquatic systems (Gruber, 2008). Understanding the dynamics of N cycling, including the factors that control individual processes and the organisms involved, has been a major goal of microbial ecologists and biogeochemists for decades. The information presently available has been used to develop global models aimed at further understanding how these dynamics affect community structure and the cycling of other key elements, particularly carbon (C) (Galloway et al., 2008). Ultimately an overarching goal is resolving the extent primary production is driven by new vs. regenerated sources of N so this can be used to further refine these models (Falkowski et al., 1998).

Subsets of microbes contribute to both autotrophic and heterotrophic processes in aquatic freshwater and marine systems. The canonical view of this is that photoautotrophic protists and cyanobacteria are important primary producers while heterotrophic microbes participate primarily in the degradation and remineralization of this newly formed organic material (Kirchman, 1994). Extensive work has shown that diverse clades of microorganisms, e.g., SAR11 and *Prochlorococcus*, may transition between subgroups of phylogenetically similar organisms that possess distinct physiological characteristics (i.e., ecotypes) based on fluctuations in their physical and chemical environment (Rocap et al., 2003; Johnson et al., 2006; Carlson et al., 2009; Galand et al., 2009, 2010; Vergin et al., 2013). Shifts in the predominance of these specific ecotypes may affect the patterns and regulation of key environmental processes (Rocap et al., 2003; Johnson et al., 2006; Carlson et al., 2009; Galand et al., 2009, 2010; Vergin et al., 2013), e.g., the production and degradation of organic material. However, the dynamics of these interactions are not well understood reinforcing the importance of characterizing the roles of different ecotypes in the environment in order to constrain their effect.

The identification and characterization of key players in microbial communities were, until recently, largely restricted to the correlation of molecular biological observations (e.g., the presence of functional genes or transcripts) with biogeochemical or physiological measurements (Zehr et al., 2001; Montoya et al., 2004; Lam et al., 2009) and the cultivation of isolates. However, most microorganisms in the environment resist current culturing techniques while those isolated are often minor parts of the community that are selected for based on the isolation method (Staley and Konopka, 1985). Strain-specific genetic and functional differences can also impede the disentanglement of an individual’s niche and how a specific ecotype may fit into a complex ecosystem. Traditional bulk assays may only access the most active and abundant organisms during the time of sampling, potentially missing important but relatively rare or ephemeral taxa (Sogin et al., 2006). Such information can provide a foundation for future work, but can be misleading if not interpreted cautiously since this may only supply a superficial glimpse into the potential metabolic diversity and potential of an ecosystem.

**FIGURE 1:**
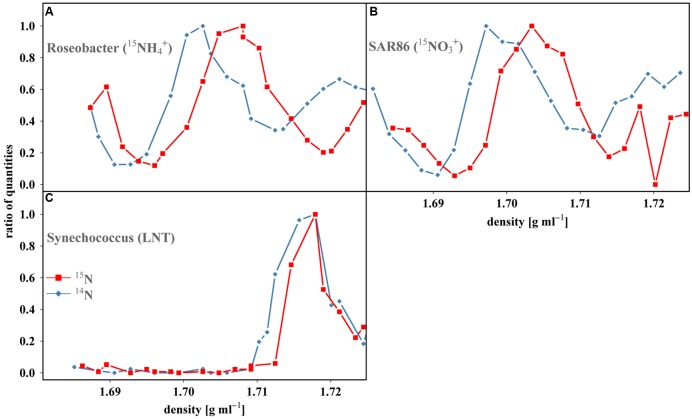
**Tag-SIP profiles of OTUs from each treatment resolved within a CsCl gradient demonstrating its ability to assess density shifts.** Each point represents a single fraction within the density gradient and the number of reads quantified within each fraction were normalized as a function of maximum number of reads in each gradient (ratio of quantities). A definitive density shift can be seen for both **(A)**, *Roseobacter* (incubated with ^15^NH_4_^+^) and **(B)**, *SAR86* OTUs (^15^NO_3_^-^) reflecting the uptake and assimilation of ^15^N-labeled substrate, while no discernable shift can be observed in **(C)**, *Synechococcus* in the Low Nitrate Treatment (LNT). Blue diamonds represent control fractions (^14^N-substrate amendments). Red squares represent treated fractions (^15^N-substrate amendments).

**FIGURE 2:**
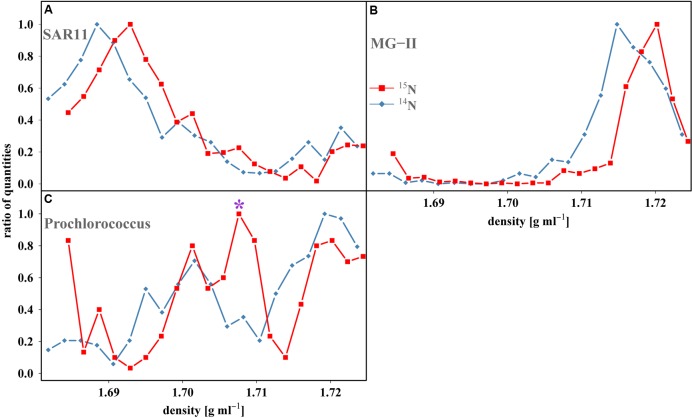
**Tag-SIP profiles of OTUs from the nitrate treatment resolved within a CsCl gradient in order to assess density shifts.** Each point represents a single fraction within the density gradient and the number of reads quantified within each fraction were normalized as a function of maximum number of reads in each gradient (ratio of quantities). Clear density shifts are easily observed for both **(A)**, *SAR11* and **(B)**, *MG-II* OTUs while the enrichment of the **(C)**, *Prochlorococcus* OTU is less apparent. **(C)**, The bimodal density distribution found in the isotopically treated sample (red squares) is likely produced by a more active subset of the population that is assimilating or replicating at a faster rate relative to the rest of the population. This would increase the amount of label within this subpopulation’s DNA and cause the formation of a heavier secondary peak (designated by ^∗^). We are confident this is a true secondary peak since it is comprised of three distinct fractions. Blue diamonds represent control fractions (^14^N-substrate amendments). Red squares represent treated fractions (^15^N-substrate amendments).

Stable isotope probing (SIP) has gained popularity in recent years due to its ability to provide direct evidence of substrate assimilation by specific taxa, thereby revealing their functional roles within a community (Neufeld et al., 2007a; Buckley et al., 2008; Addison et al., 2010; Nelson and Carlson, 2012). Actively growing organisms capable of assimilating substrates enriched with ^13^C or ^15^N incorporate these heavy isotopes into newly formed nucleic acids, increasing the density of these molecules relative to those same organisms in controls grown on ^12^C or ^14^N-substrates (Neufeld et al., 2007b). The nucleic acids are then separated by density gradient ultracentrifugation and a variety of techniques can then be employed to both detect this enrichment and identify the organisms assimilating the substrate of interest. Uptake of ^15^N-substrates can result in a maximum density increase of only ~50% of that achievable with ^13^C-substrates (Buckley et al., 2007a). An additional complication in interpretation of N SIP data is the range of DNA GC-content (Buckley et al., 2007b) found within a sample, making density shifts, particularly in individual species, difficult to differentiate. Offsetting many of these problems, Tag-SIP (16S rRNA gene amplicon or ‘tag’ sequencing, combined with SIP) increases the resolving power of traditional SIP by examining the densities of individual OTU’s DNA to more easily identify and compare shifts (**Figures 1** and **2**), similar to a recent study (Connelly et al., 2014).

Tag-SIP was applied to coastal surface waters in order to investigate our hypothesis that a population’s capacity to assimilate different N sources, particularly on the subgroup level, is much more diverse than is currently assumed, ultimately leading to an incomplete understanding of community structure, functional capacity, and metabolic activity. We provide evidence of intraclade heterogeneity with respect to both ammonium and nitrate assimilation, shedding light on the activity of individual organisms and the roles they may play in the cycling of nutrients *in situ* (Fuller et al., 2005; Follows et al., 2007).

## Materials and Methods

### Water Sampling and ^15^N-Isotope Experiments

Surface water was collected on 06 August at dawn and the early evening and 01 September in the early evening from Big Fisherman’s Cove off Catalina Island, Avalon, CA, USA, by bucket for treatment Ammonium, Nitrate, and Low Nitrate (LNT). This water was distributed into 2L acid washed high density polyethylene bottles after three rinses with ambient sea water. Samples were amended with either ^15^N labeled ammonium or nitrate and control samples of ^14^N-substrate (Sigma-Aldrich, USA) were incubated in parallel (2 μM was used in August and 0.2 μM was used in September). Bottles were submerged in the same waters as the collection site in bags composed of two layers of neutral density screening to simulate ambient average light intensity and temperature for 24 h. Subsamples were also collected, filtered through 0.2 uM Supor membrane Acrodisc syringe filters (Pall Life Sciences, USA), and stored in 50 ml centrifuge tubes (VWR, USA) in -20°C freezer for later processing of nutrient measurements. Incubations were terminated by peristaltic filtration onto 0.2 μm Supor filters (Pall Life Sciences, USA), immediately flash frozen in liquid N, and stored under -80°C until extraction in the laboratory.

### DNA Extraction and CsCl Gradient Ultracentrifugation

DNA was extracted using DNeasy kit (Qiagen, USA) with additional liquid N freeze/thaws and bead beating (30 s) prior to extraction. DNA was quantified with the Qubit assay (Invitrogen, USA) and ~750 ng of DNA from each samples was added to separate CsCl gradient and centrifuged in a Beckman (USA) NVT65.2 rotor at ~44 krpm for ~66 h at 20°C. Upon completion of centrifugations, gradients were fractioned and purified based on a modified protocol (Neufeld et al., 2007b). Individual gradients were displaced by mineral oil and collected as fifty ~100 μl fractions. Density was determined on each fraction using a modified AR200 handheld digital refractometer (Reichert, USA) as described by Buckley et al. (2007b). DNA was then precipitated in all fractions after the addition of two volumes of 30% polyethylene glycol (PEG) solution and 20 μg glycogen followed by a 70% ethanol wash.

After elution in 30 μl of TE buffer, the distribution of DNA in the CsCl gradient was determined through the quantification of each fraction using the Qubit assay (Invitrogen, USA). This information was used to select 20 fractions from each treatment from both isotopically labeled and control samples for further processing. PCR was carried out on each of these fractions using fusion primers that amplified a segment of the V4V5 region of the 16S rRNA gene (515F 5′-GTGYCAGCMGCCGCGG and 926R (5′-CCGYCAATTYMTTTRAGTTT) which allowed multiplexing through the use of an inline five base pair (bp) barcode on the forward primer and a unique 6 bp index on the reverse primer (Huse et al., 2014). PCR conditions and potential organisms missed by these primers are discussed extensively in Parada et al. (2016). These amplicons were sequenced on the Illumina MiSeq platform at the University of California, Davis sequencing core. They were demultiplexed with QIIME (Caporaso et al., 2010) and Mothur (Schloss et al., 2009), quality filtered using UPARSE (Edgar, 2013) and Mothur (Schloss et al., 2009), chimeras were removed using UCHIME (Edgar et al., 2011), and clustered into OTUs (at 99%) using the average neighbor algorithm in Mothur (Schloss et al., 2009). Taxonomy was assigned with Mothur (Schloss et al., 2009) using the SILVA database (Quast et al., 2013) available on the Mothur wiki at the time this data was analyzed. The top 100 OTUs were then individually assessed to determine the position of DNA banding in both treated and controlled samples. Only OTUs with at least ~400 reads per sequenced fraction were considered in this initial analysis in order to minimize potential artifacts such as those that may be caused by random banding of exceedingly low abundance organisms. Those that showed shifts toward the denser part of the gradient relative to the control were considered labeled with isotope and therefore actively assimilating the substrate tested. Genomic data have been deposited in the European Nucleotide Archive under accession numbers ERS980492–ERS980611.

### Nutrient Analysis

Ammonium was measured in triplicate using the OPA method and was corrected for matrix effects, limit of detection of ~0.03 (Taylor et al., 2007). Samples were also analyzed in triplicate at the Marine Science Institute Analytical Laboratory at University of California, Santa Barbara for both phosphate and nitrite + nitrate by standard colorimetric methods (Parsons et al., 1984). Limits of detection are approximately 0.03, 0.05, and 0.1 μM, respectively.

### Phylogenetic Analysis

16S rRNA gene sequences of SIP OTUs of interest were aligned in Geneious (Kearse et al., 2012) with related publically available sequences obtained from GenBank (Benson et al., 2015) with ClustalW (Thompson et al., 2002). The K80 model (Kimura, 1980) was used to construct a maximum-likelihood tree with PhyML (Guindon et al., 2010), which were bootstrapped 1000 times.

## Results

### Identifying Enrichment and the LNT

To better assess the consistency of DNA banding patterns within a gradient, a 10-fold lower amendment of nitrate, the Low Nitrate Treatment (LNT), was undertaken. The ratio of the added substrate concentration: ambient concentration (C_s_:C_a_) was ~0.67 and the atom% ^15^N of the available nitrate pool after the addition of the ^15^NO_3_^-^ amendment <40% (**Table 1**). Assuming a single doubling occurred during the 24 h incubation and nitrate was the sole N source for growth, the maximum incorporation of isotope by any OTU during this incubation would have been expected to be <20%, due to the semiconservative nature of DNA replication. The LNT OTUs likely have little to no detectable incorporation of isotope based on previous SIP studies [≥30 atom% enrichment, (Murrell and Whiteley, 2011; Connelly et al., 2014)], so differences between the position or density of an individual control and treated OTU can likely be attributed to methodological variations in DNA banding, i.e., the method’s ability to resolve the position or density of an individual DNA fragment or OTU. This information can then be used to help set the identification criteria for density shifts.

**Table 1:** Comparison of C_s_:C_a_ across each treatment.

Treatment	Phosphate [μM]	Ammonium [μM]	Nitrate [μM]	C_s_^c^ [μM]	(C_s_:C_a_)^d^	Atom% ^15^N enrichment^b^	Percentage of OTUs with uptake
Ammonium	0.26 ± 0.016	700.72 ± 0.030	1.19 ± 0.059	2.0	2.78	72.2%	47%
Nitrate	0.27 ± 0.0047	1.19 ± 0.034	700.47 ± 0.012	2.0	4.26	79.4%	62%
LNT^a^	0.16 ± 0.0034	0.84 ± 0.15	700.30 ± 0.011	0.2	0.67	39.4%	0%

*^a^Low nitrate treatment. ^b^Referring to the combined atom% ^15^N of the available N pool for the substrate of interest during the incubation. ^c^Concentration of isotopically labeled N spike. ^d^Concentration of ambient nutrient being assessed. Ambient concentrations of phosphate, nitrate, and ammonium and additions. C_s_ is the final substrate concentration of the spike added to sample. C_a_ is the ambient concentration of the substrate being probed and is designated for each treatment by orange shading. Concentrations are reported in micrometer with ± 1 SD.*

Reads from control and isotopically treated samples largely banded at the same density and had a similar overall shape (Supplementary Figure S1). This generally overlapping symmetry between these bands was the most convincing evidence of the validity of the method, demonstrating that unlabeled DNA (including DNA that was labeled below the detection limits of the method) banded in a reproducible fashion for each individual OTU (Supplementary Figure S1). Nearly all density differences between individual control and treated OTUs were near zero (~90% had shifts ~0–0.001 g ml^-1^) while only five pairs reached the largest density difference recorded in the LNT of 0.002 g ml^-1^ (*n* = 100, mean = 0.0004, *SD* = 0.0007). Conservatively we chose a value 1.5x the largest shift, i.e., 0.003 g ml^-1^ which is ~4 SD from the mean, to designate a sample enriched relative to its control, in an attempt to avoid the potential of false positives. The Ammonium and Nitrate treatments were then examined and a large portion of OTUs had shifts ≥0.003 g ml^-1^ (47 and 62% for Ammonium and Nitrate, respectively, Supplementary Table S1; **Figures 1A,B** and **2**), likely reflective of the much higher (C_s_:C_a_) and atom% ^15^N found within these treatments (**Table 1**). It should also be noted that all density shifts were positive, i.e., if there was a density shift, the isotopically treated OTU was always heavier than its control OTU. Negative shifts, i.e., where the control OTU is identified as heavier than the isotopically treated OTU, would potentially occur if the position of DNA banding within a gradient was not consistent nor a factor of its density.

### Heterotrophic Nitrate Assimilation and Intraclade Heterogeneity

We directly detected multiple, putatively heterotrophic bacterial clades with a large percentage of members demonstrating nitrate uptake (e.g., 44% of γ*-Proteobacteria*, 90% of *Flavobacteriaceae*, and 59% of *α-Proteobacteria*, Supplementary Table S1). Overall the OTUs found within specific heterotrophic bacterial clades identified with evidence of heavy isotopic incorporation ranged from 20–100% and 0–100% for nitrate and ammonium, respectively (**Table 2**). Notably, not all the OTUs within these clades displayed the same level of enrichment, in fact some showed no detectable enrichment. This intraclade heterogeneity was seen within almost every heterotrophic clade assessed (**Table 2**; Supplementary Figures S4 and S5), demonstrating this result may be potentially widespread among these marine microbial populations. This lack of an all or nothing strategy with respect to ^15^N assimilation found within the Nitrate treatment points to the potential harboring of some members that may be metabolically flexible.

**Table 2:** Select clades and their percentages of OTUs demonstrating uptake within each treatment.

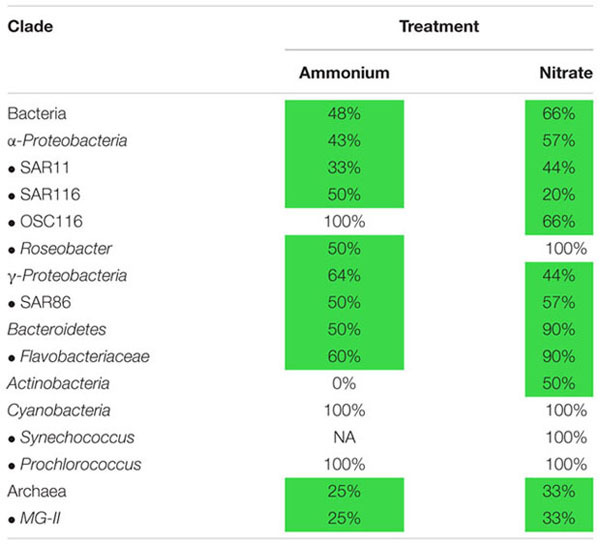

*Green shading signifies heterogeneity within that specific clade with respect to the substrate listed. Low Nitrate (LNT) was omitted from this table because no uptake was found within treatment. NA mean not analyzed and differs from 0% in that it signifies that OTUs from this clade were not recovered within the top 100 OTUs from this treatment and therefore were not analyzed.*

**FIGURE 3:**
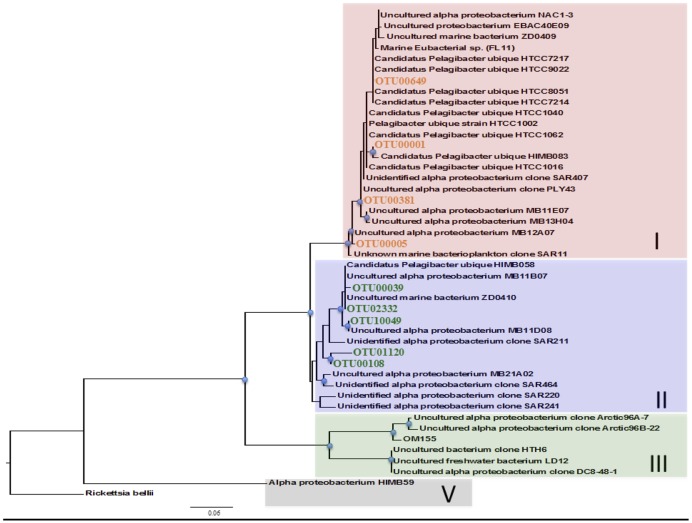
**Phylogenetic divide among *SAR11* OTUs with respect to nitrate uptake.** Maximum-likelihood 16S rRNA phylogenetic tree of *SAR11* OTUs from the nitrate treatment using PHYML (PHYlogenetic inferences using Maximum-Likelihood) with 1000 bootstraps. OTUs identified as assimilators (orange text) are all found within subclade I whereas those without isotopic enrichment (green text) resided within subclade II. Phylogenetic clade/ecotype designated at the right. The scale bar represents 0.06 substitutions per site. Blue nodes indicate bootstrap values greater than 50. *Rickettsia bellii* was used as the outgroup.

One clade of particular interest was the cosmopolitan SAR11 where 44% of OTUs exhibited evidence of nitrate assimilation (**Figure 2A**; Supplementary Figure S4). To the best of our knowledge, while no genes directly involved in nitrate assimilation have been recovered in culture or environmental studies of SAR11, our work is the first evidence of this potential capability within this clade. Additionally, nitrate assimilation appears to follow phylogenetic lines as all OTUs that did not assimilate nitrate were found within subclade II, while those with enrichment are from subclade I (**Figure 3**). These differences likely reflect variations in metabolic activity and capacity among these closely related organisms and potentially suggest niche partitioning among ecotypes.

In contrast, all members of the autotrophic clades analyzed, i.e., the cyanobacteria *Prochlorococcus* and *Synechococcus*, demonstrated the ability to assimilate nitrate, and all *Prochlorococcus’* were active with respect to ammonium (**Table 2**; Supplementary Figure S2). However, the total number of cyanobacterial OTUs recovered were much lower than that of the heterotrophic clades and no *Synechococcus* OTUs were analyzed for ammonium activity, so little can be said regarding intraclade heterogeneity within the autotrophic cyanobacteria from in this study (**Table 2**).

### Intraclade Heterogeneity for Nitrate Uptake within an Archaeal Clade

Functional heterogeneity with respect to potential nitrate uptake was not only found in bacterial taxa, but also among the euryarchaeal MG-II (**Figure 2B**; Supplementary Figure S5), which was the only member of the Archaeal domain analyzed in this study. These results ranged from 25–33% identified as positive assimilators and were the lowest non-zero range found for any clade or domain assessed. The OTUs displaying density shifts were related to members of subclade IIa, while the OTUs without isotopic enrichment resided in subclades IIa and IIb (Supplementary Figure S6).

## Discussion

DNA recovered from natural populations of prokaryotic assemblages was used to assess a community’s capacity to incorporate a particular isotopically enriched N-substrate. This was accomplished through the identification of a relative increase in density between DNA bands of OTUs recovered from control and isotopically treated samples using the Tag-SIP methodology. In order to establish the density of each OTU, the peak of the DNA band had to be contained in at least three fractions to be assessed, while single point peaks were ignored. In the rare occasion where no definitive peak fitting the criteria was observed, the overall distribution of the bands was compared. Previous methods using similar criteria have shown this general approach to be valid (Buckley et al., 2007a,b; Wawrik et al., 2009; Nelson and Carlson, 2012; Wawrik et al., 2012; Connelly et al., 2014). However, a limit of detection needed to be tested before enrichment could be properly identified in our study.

Based on the results of the LNT, a limit of detection was conservatively set at 0.003 g ml^-1^. Additionally, DNA repair can increase the overall incorporation of label into DNA without any subsequent cell divisions (Alberts et al., 2002) and taxa with higher metabolic rates could potentially carry out more than a single cell division during these incubations. This potentially increases the probability that some OTUs within the LNT were labeled, further bolstering this as a conservative cutoff for enrichment. Therefore, we are confident that any shifts in DNA equal to or greater than this limit of detection were due to the active assimilation of labeled substrate making this value more sensitive than those reported previously [>30%, (Murrell and Whiteley, 2011; Connelly et al., 2014]. This cutoff was then applied to the ammonium and nitrate treatments to evaluate these communities’ ability to assimilate different N sources.

Not all OTUs displayed evidence of ammonium assimilation during the incubation (**Table 2**) although it is generally assumed that most microbes can carry out this process. Thus, this heterogeneity with respect to ammonium assimilation may actually tell us more about the activity of these OTUs than their functional capacity. This is because SIP is not just testing capacity but also activity in general. Many factor can play into the activity and labeling of individual microbes *in situ* including life history and the environmental conditions, both of which can greatly affect their physiological state (Blazewicz et al., 2013) leading up to and during the incubation period. This can be compounded by taxon diversity where OTUs may possess distinct genetic or physiological machinery that may differ in transcription rates, kinetic effects caused by substrate concentration, or mechanism of regulation (Vergin et al., 2007; Berges and Mulholland, 2008; Tripp et al., 2008). Dormant or dead cells will have little chance of ammonium assimilation, but their DNA can still be recovered and assessed. Conversely, active utilization of the substrate can occur without simultaneous or subsequent DNA turnover. Both situations would lead to a negative result; SIP labeling only occurs after the substrate is taken into the cell and incorporated into DNA at a fast enough rate to significantly increase the density of their DNA above the limit of detection over the incubation period. An isotopically labeled OTU must almost certainly be active in order for this labeling to occur. SIP therefore may be more sensitive in identifying the most active fraction of a mixed community than more standard methods, e.g., rRNA analyses, since there is not always a direct correlation between activity and rRNA concentration (Gausing, 1977; Blazewicz et al., 2013). The OTUs labeled within the ammonium treatment likely represent the most active members of the microbial population during the time of sampling (**Table 2**).

A diverse array of heterotrophic clades demonstrated significant evidence of nitrate utilization. These results directly complement previous work (Allen et al., 2001; Cai and Jiao, 2008) which had uncovered *nasA* genes (assimilatory nitrate-reductase) within diverse groups of heterotrophic bacterial clades distributed among contrasting oceanic regimes, and bacterial uptake of nitrate accounted for up to 40% of the total nitrate uptake at five stations in the Barents Sea (Allen et al., 2002). In more recent investigations, culture dependent and independent analyses of diverse marine environments suggested that previously surveyed *nasA* genes had been underestimated (Jiang et al., 2015) while Wawrik et al. (2012) provided evidence of nitrate assimilation by heterotrophs despite the inability to recover transcripts of *nasA* genes. These results further demonstrate that heterotrophic organisms can and are actively competing with the phototrophic community for this oxidized N source.

Initial genomic surveys and culture work reported *Prochlorococcus*’ inability to assimilate nitrate (Moore et al., 2002), but more recent studies have indicated otherwise (Casey et al., 2007; Martiny et al., 2009; Treibergs et al., 2014). Our direct evidence suggests, by way of the presence of prominent, shifted secondary peaks (**Figure 2C**; Supplementary Figure S2) that assimilation of nitrate by *Prochlorococcus* occurs *in situ*. Secondary peaks are not uncommon in SIP (Buckley et al., 2007a; Wawrik et al., 2009) and likely point toward the presence of subpopulations within an individual OTU with different levels of activity or nutrient pathways, which has been proposed previously for this clade (Martiny et al., 2009; Biller et al., 2014; Kashtan et al., 2014). Both OTUs recovered showed enrichment with similar banding patterns providing potential validation of nitrate utilization in this study (Supplementary Figure S2). Phylogenetic analysis reveals that our *Prochlorococcus* OTUs fall within the high-light (HL) ecotype (Supplementary Figure S3) and are most similar to the MED 4 strain (100 and 99.7%).

Our results are supported by the work of Martiny et al. (2009) who showed a genomic island, or irregular regions of the genome often used to identify possible sites of lateral gene transfer (LGT), containing *Synechococcus*-like genes involved in nitrite and nitrate acquisition. These genes were also found to code functional proteins and their distribution was shown to be widespread in marine surface waters. Furthermore, a recent study uncovered genes involved in nitrate assimilation within three distinct *Prochlorococcus* lineages, as well as the growth of isolates solely on nitrate, including members of the HL ecotype (Berube et al., 2015a). Berube et al. (2015a) only recovered these genes in HLII, while our study suggests nitrate utilization in members of HLI but no members of any other subclade were observed or analyzed within the top one hundred OTUs. This would be expected as our study site contains colder waters with higher nutrients where HLI would be likely to dominate (Johnson et al., 2006) compared to the much warmer waters where most of their strains were isolated (Berube et al., 2015a). They suggested that genes for nitrate assimilation may have been gained and lost multiple times as the *Prochlorococcus* clade has evolved but also had evidence that these genes may be susceptible to LGT (Berube et al., 2015a). Berube et al. (2015a) speculate that this could facilitate the introduction of this ability into other lineages.

These studies, in combination with our evidence of *in situ* nitrate utilization, confirm that this cyanobacterial clade is much more physiologically and genetically diverse than originally perceived (Moore et al., 2002; Kettler et al., 2007). *Prochlorococcus* is the most abundant phototroph on the planet (Johnson et al., 2006; Flombaum et al., 2013), and so any changes in the understanding of this clade’s physiology or ecological niche will improve our current perspective of its role in the microbial ecology of the ocean (Rocap et al., 2003; Giovannoni and Stingl, 2005; Johnson et al., 2006; Martiny et al., 2009). Our understanding of N cycling and new production in oligotrophic area, such as the tropical and subtropical open oceans, will likely be affected most since *Prochlorococcus* can account for up to ~45% of the photosynthetic biomass and carry out a significant fraction of net primary production (~10–50%) in these regions (Goericke and Welschmeyer, 1993; Liu et al., 1997). Maximum abundances are observed when surface waters become stratified and nutrients are depleted, increasing the competition for nitrate. A recent study carried out in the Atlantic and Pacific attempted to examined this by quantifying nitrate assimilation genes within the HLII and found them to be 20–50% of the population under stratified conditions (Berube et al., 2015b). Further work is required to understand the spatial and temporal variations of these nitrate utilizing ecotypes in order to better assess and appreciate the role *Prochlorococcus* plays in the marine community as well as in both N and C cycling.

Evidence of nitrate assimilation within the SAR11 clade appears to follow phylogenetic lines as all OTUs that did not show evidence of nitrate assimilation were found within subclade II, while those with enrichment were from subclade I (**Figure 3**). This suggests potential niche partitioning among ecotypes, wherein subpopulations’ distinct phenotypic variations are selected for when certain environmental conditions develop. The generally higher-nutrient coastal waters investigated in this study may be more likely to harbor taxa with the genetic capacity to respond to N sources across a range of oxidation states. Our nitrate amendment may have enriched for this particular ecotype, facilitating its detection. Ecotypes are not uncommon within the SAR11 clade and previous work has suggested it has evolved into at least a dozen specialized ecotypes who’s relative abundances often appear to correlate with fluctuations in environmental parameters (Carlson et al., 2009; Vergin et al., 2013). Many of these subclades harbor hypervariable regions that have been implicated in the acquisition of novel genetic material, enabling strain-specific metabolisms, e.g., sulfur metabolism unique to strain HTCC9565 of SAR11 (Grote et al., 2012). A recent study investigating single amplified genomes (SAG) off the coast of Mexico uncovered the genetic potential to reduce nitrate within several subclades of SAR11 taken from the oxygen minimum zone (Tsementzi et al., 2016). Though assimilatory pathways were not address directly in their study, genes involved in both the uptake and reduction of nitrate were present and actively transcribed (Tsementzi et al., 2016). This result in combination with our direct evidence further bolsters the potential that an assimilatory pathway is present and active within the SAR11 clade. Tag-SIP may facilitate a fine scale look at the functional capacity of individuals, analogous to SAG, through the use of targeted N amendments and fractional gradient sequencing.

Nitrate assimilation may have only recently arisen within the SAR11 lineage when subclades I and II diverged (**Figure 3**). Acquisition of this capability may have contributed to the divergence. Alternatively, microdiversity may have independently developed multiple times. The physiological state or life history of the community at the time of sampling may have also played a role in these apparent intraclade differences, potentially making these results more of a reflection of increased activity than lack of capacity, as mentioned previously. These differences in activity or capacity could potentially help explain some of the aforementioned variations in abundance and distribution patterns often associated with this clade (Carlson et al., 2009; Vergin et al., 2013). Therefore, a complete understanding of the dynamics of SAR11’s metabolic potential is essential to fully understanding its role in organic C degradation and sequestration in the oceans, particularly in the vast expanses of the oligotrophic gyres where nutrients are low and SAR11 abundances are high (Morris et al., 2002; Carlson et al., 2009; Brown et al., 2012; Vergin et al., 2013).

Similar heterogeneity was observed within many other major bacterial clades including *Roseobacter*, SAR86, and *Flavobacteriaceae* (**Table 2**), which we see as additional evidence of phenotypic variation of closely related organisms and potentially ecotypes exhibiting different metabolic strategies, and/or activities, allowing them to occupy discrete environmental niches. This result was not only restricted to the bacterial domain but was also found within the archaeal clade MG-II. Previous work surveying 16S rRNA genes similarly uncovered potential evidence of functional differences among these subclades (Galand et al., 2009, 2010). However, no cultured representatives exist from MG-II, so there is little known about their modes of nitrogen metabolism, although the ability to both transport and utilize nitrate through dissimilatory and assimilatory pathways has been found widespread throughout the Archaeal domain (Cabello et al., 2004). A single recently closed genome representing multiple strains of MG-II did not appear to contain any known genes associated with nitrate utilization (Iverson et al., 2012) and no cultured representatives currently exists. Since ours is the first dataset of this nature for MG-II, which often dominates archaeal gene surveys in the marine water column (DeLong et al., 2006; Galand et al., 2009), a reconsideration of the role of archaea in the uptake of N is indicated, just as we have had to revise our conceptual model to include a role of Archaea in ammonium oxidation (Wuchter et al., 2006).

It is important to acknowledge the possibility that some of our nitrate results may be affected by cross-feeding, where uptake of labeled nitrate by one organism is then metabolized to another form, e.g., ammonium, released, and assimilated by another organism yielding a false positive. We argue that nitrate uptake, metabolism, and release of sufficient substrate by one organism, in close enough proximity to another organism that is able to take it up and enrich their DNA by the ~20% required for detection, within a 24 h incubation, is a highly unlikely, particularly given the dilution of the enriched substrate by ambient pools of unlabeled nitrogen. A highly sensitive direct visualization technique, i.e., nanoSIMS, showed that, despite being attached directly to an actively fixing nitrogen fixer (diazotroph), epibiotic (i.e., attached) heterotrophic bacteria were only weakly enriched secondarily with released ^15^N, and cells that were <10 μm away from the diazotroph showed no observable enrichment (Behrens et al., 2008).

Though rates were not measured directly in this study, measurements in a previous investigation within the Southern California Bight (SCB) can be used to further demonstrate the unlikelihood of cross-feeding here. Bronk and Ward (2005) reported nitrate uptake rates of ~0.01 μmol N L^-1^ h^-1^ that would equate to an ~20% enrichment of the ~1.25 μM N PON. This enriched PON could theoretically produce ~0.009 μM of ^15^N-DON and ~0.13 μM of ^15^N-NH_4_^+^ during this incubation with release rates of ~0.002 and 0.028 μmol N L^-1^ h^-1^, respectively (Bronk and Ward, 2005). If these were the sole ^15^N sources being released, this would make up a small fraction, i.e., ~8%, of the total N taken up through ammonium assimilation based on the rates measured in the SCB study [~0.072 μmol N L^-1^ h^-1^; Bronk and Ward, 2005] and this enrichment would be undetectable by Tag-SIP. Furthermore, the relatively high concentration of unlabeled ambient ammonium (1.19 μM, **Table 1**) and DON [~5–7 μM; Bronk and Ward, 2005] in these waters would dilute any labeled ammonium or DON released, additionally decreasing the possibility of measurable cross-feeding. Therefore, it is likely that only the most metabolically active organisms with consistent access to the primary labeled substrate would be distinguishable from the rest of the community with our method. Finally, in all treatments, the percentages of DNA labeling found in organisms that are thought to be more active, e.g., phototrophs, were similar to those found in the presumably less active heterotrophic community (data not shown), possibly reflecting use of the same labeled source. Therefore cross-feeding was considered highly unlikely to have any major effects on the results.

If any cross-feeding was occurring, it would probably come from highly active organisms living in very close association with primary utilizers. Their metabolic activities would have to be tightly coupled in order to accumulate enough secondary label within these 24 h incubations. This type of cross-feeding would still be quite informative, delineating an effective and rapid trophic cascade. Such results would potentially be evidence of some type of microbial association, e.g., symbiosis, where nutrients were rapidly transferred directly in at least one direction, though the mechanism would remain unclear. Alternatively, no specific associations could be involved but the taxa involved would have to be specifically geared toward scavenging newly produced N quickly and efficiently. These populations may continuously be maintaining significant amounts of resource-acquisition machinery primed and ready or investing heavily in growth machinery to facilitate rapid growth when fluxes of regenerated N occur, similar to the optimization model of phytoplankton (Klausmeier et al., 2004). These tightly coupled associations in combination with the rapid and efficient exploitation of freshly released N could potentially play a role in the global dominance seen in heterotrophic clades like SAR11 and MG-II, permitting them to preferentially access to exudates.

This dominant nature associated with some of the major clades, e.g., MG-II, SAR 11, and *Flavobacteriaceae*, may be further explained by the functional heterogeneity we observed, where any one member of a subpopulation may be more suited under a particular set of environmental conditions, allowing the population to weather a range of variables, e.g., changes in light or nutrients. This may also shed light on the long standing ‘paradox of the plankton,’ which is the often observed high diversity of organisms under conditions of apparent resource limitation. Each of these fluctuations in the physical and chemical environment may select for a different population or subpopulation, maintain diversity that would otherwise be filtered out by competitive exclusion (Hutchinson, 1961). Alternatively, some ecotypes may have the ability to assimilate the substrate, but their growth rate may be too low to resolve incorporation in the length of our incubations. These differences in activity may also play a role in ecotype selection.

Our results suggest that this functional heterogeneity may be more widespread in the microbial community than previously appreciated. Resolving the extent of functional heterogeneity in the environment will likely be difficult with traditional methods, as detection usually requires long-term surveys to identify the highly structured and rhythmic patterns of variation over space and time. Tag-SIP may provide a much more tractable approach to identifying ecotypes, requiring only a single sample, as well as having the added advantage of providing direct information on their function.

Our work provides insight into the uptake of a substrate by a cell, and then furthers this insight by demonstrating that the organism is metabolically active and assimilating this substrate into biomass, i.e., DNA enrichment, something very difficult to otherwise evaluate. This enabled us to investigate some prevailing perceptions, particularly in regard to heterotrophic prokaryotes’ influence (e.g., SAR11) on nitrate dynamics as well as the potential frequency of intraclade heterogeneity. Our data suggest that this phenomenon is likely much more ubiquitous than previously understood and requires greater examination. We also uncovered what is potentially the first direct evidence of *in situ* nitrate assimilation by several important marine taxa including SAR11 and MG-II. As the sophistication of global biogeochemical cycling models increases with the inclusion of specific functional groups and ecotypes (Follows et al., 2007; Follows and Dutkiewicz, 2011), the effect of addition of these dominant marine clades to nitrogen cycling sub-models could be substantial. Tag-SIP holds great promise in gaining a robust knowledge of the specific roles of important marine groups in a range of biogeochemical processes. However, further work is required to expand its use and to corroborate these findings in order to better understand how they fit into our overall perspective of global nutrient cycling.

## Author Contributions

MM and DC designed the research; MM conducted the research; MM analyzed and synthesized the data; and MM and DC wrote the manuscript.

## Conflict of Interest Statement

The authors declare that the research was conducted in the absence of any commercial or financial relationships that could be construed as a potential conflict of interest.
