# Supplementary material for: Intraclade Heterogeneity in Nitrogen Utilization by Marine Prokaryotes Revealed Using Stable Isotope Probing Coupled with Tag Sequencing (Tag-SIP)
Source: Front Microbiol. 2016 Dec 2;7:1932. doi: 10.3389/fmicb.2016.01932 (PMC5133248; doi:10.3389/fmicb.2016.01932)
Supplement: Supplementary file 1 [file Table_1.pdf]

Supplementary Table 1:

Tag-SIP OTU breakdown for each treatment. Breakdown of uptake by OTUs within clades of interest with respect to nitrate and ammonium treatments. Percentages reflect OTUs displaying positive uptake. Values in parentheses are total number OTUs for that clade and treatment.

Treatment	All OTUs	γ-Proteobacteria	Flavobacteriaceae	Cyanobacteria	Archaea	α-Proteobacteria
Ammonium	47% (100)	64% (11)	60% (30)	100% (1)	25% (4)	43% (28)
Nitrate	62% (100)	44% (16)	90% (20)	100% (6)	33% (6)	57% (30)
LNT[a]	0% (100)	0% (17)	0% (26)	0% (4)	0% (2)	0% (29)

[a]Low Nitrate Treatment
