# Supplementary figures and images for: Intraclade Heterogeneity in Nitrogen Utilization by Marine Prokaryotes Revealed Using Stable Isotope Probing Coupled with Tag Sequencing (Tag-SIP)
Source: Front Microbiol. 2016 Dec 2;7:1932. doi: 10.3389/fmicb.2016.01932 (PMC5133248; doi:10.3389/fmicb.2016.01932)

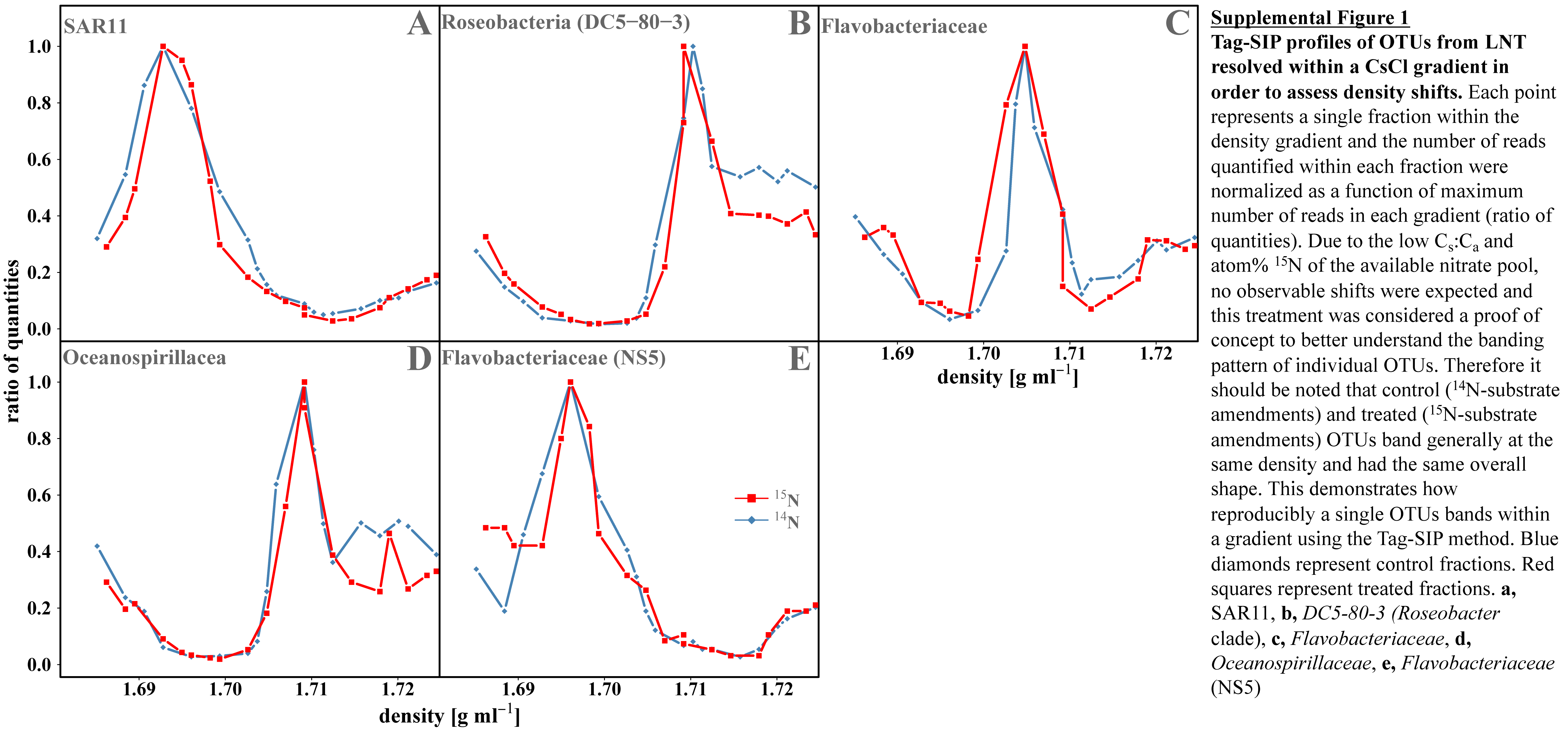

Supplement: Supplementary file 2 [file Image_1.TIF]

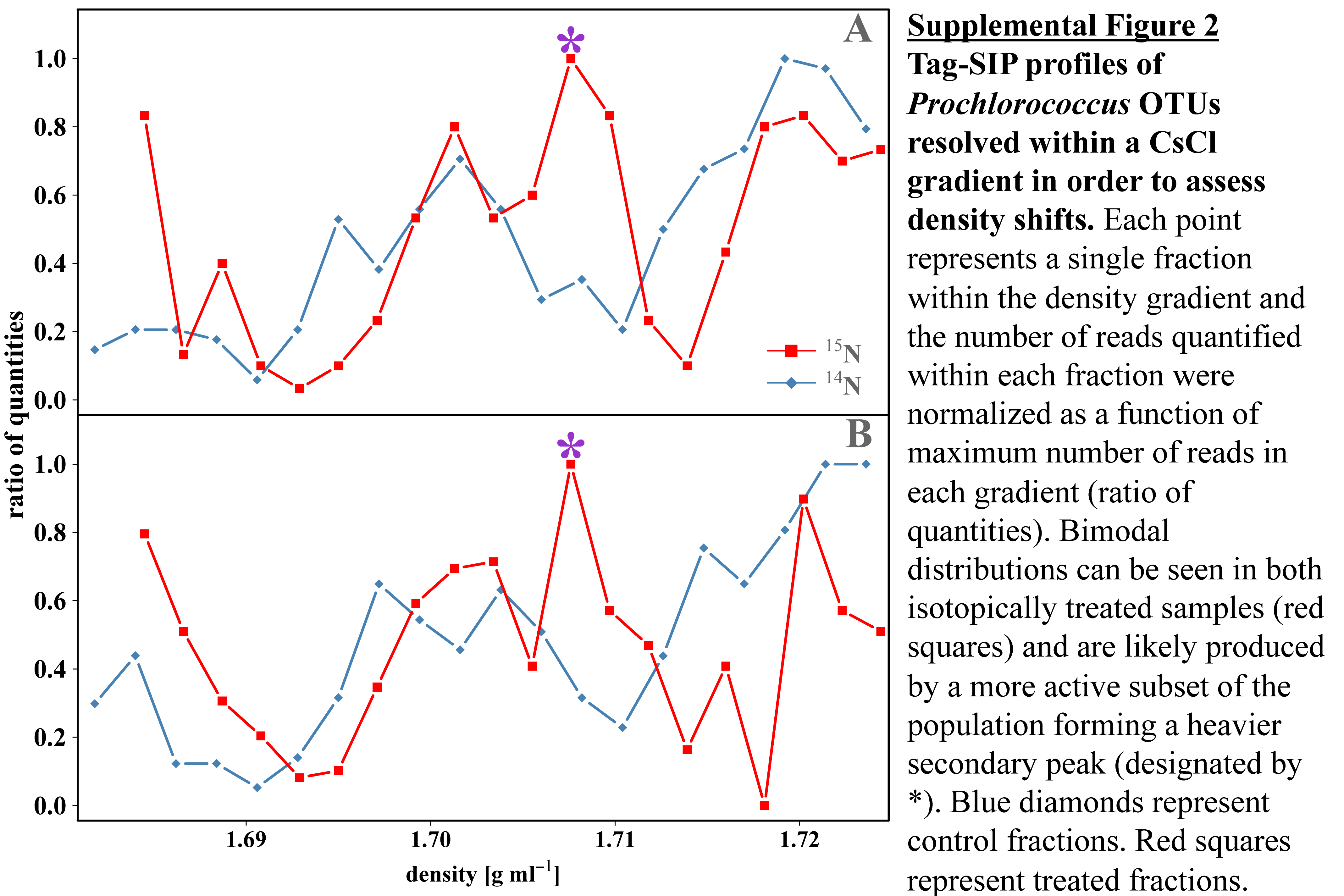

Supplement: Supplementary file 3 [file Image_2.TIF]

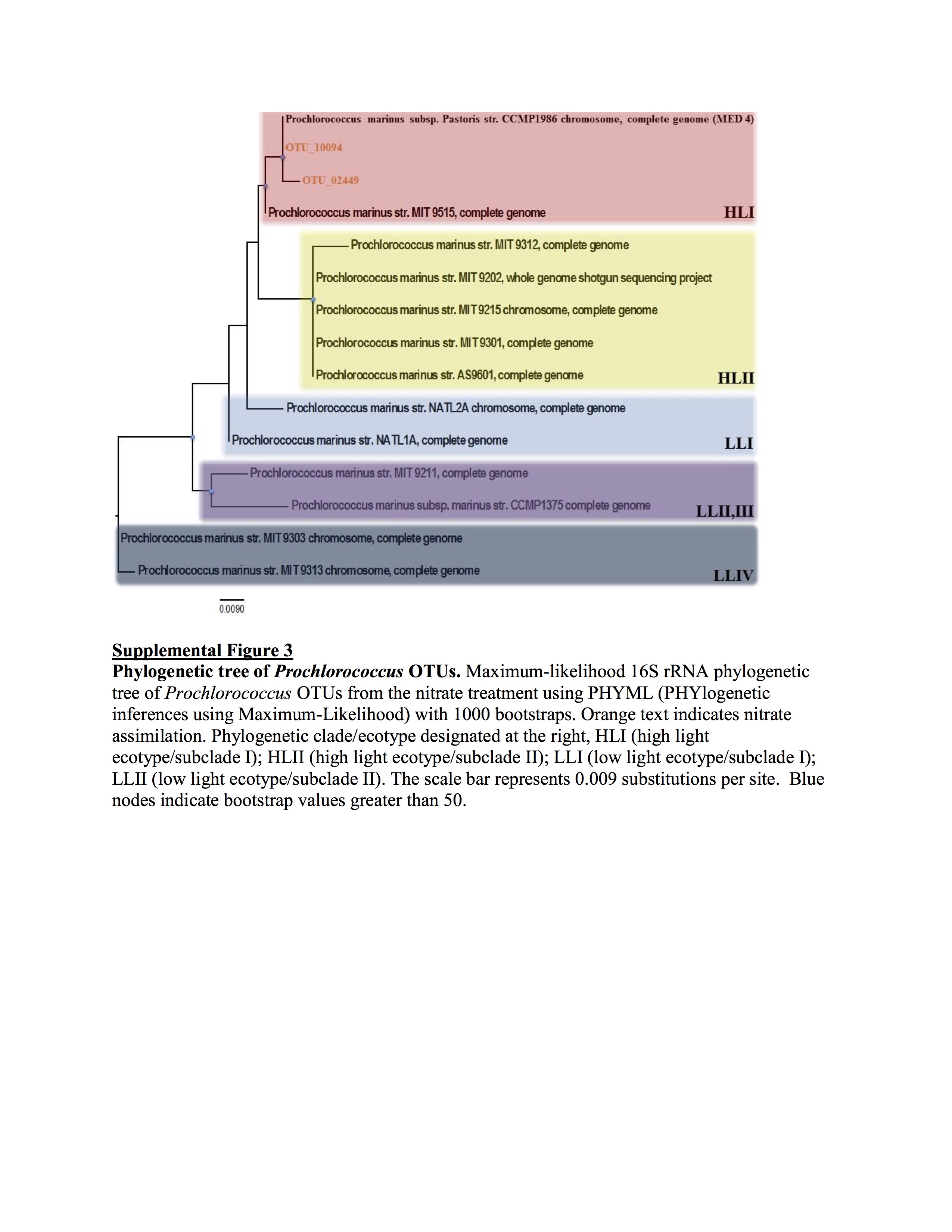

Supplement: Supplementary file 4 [file Image_3.JPEG]

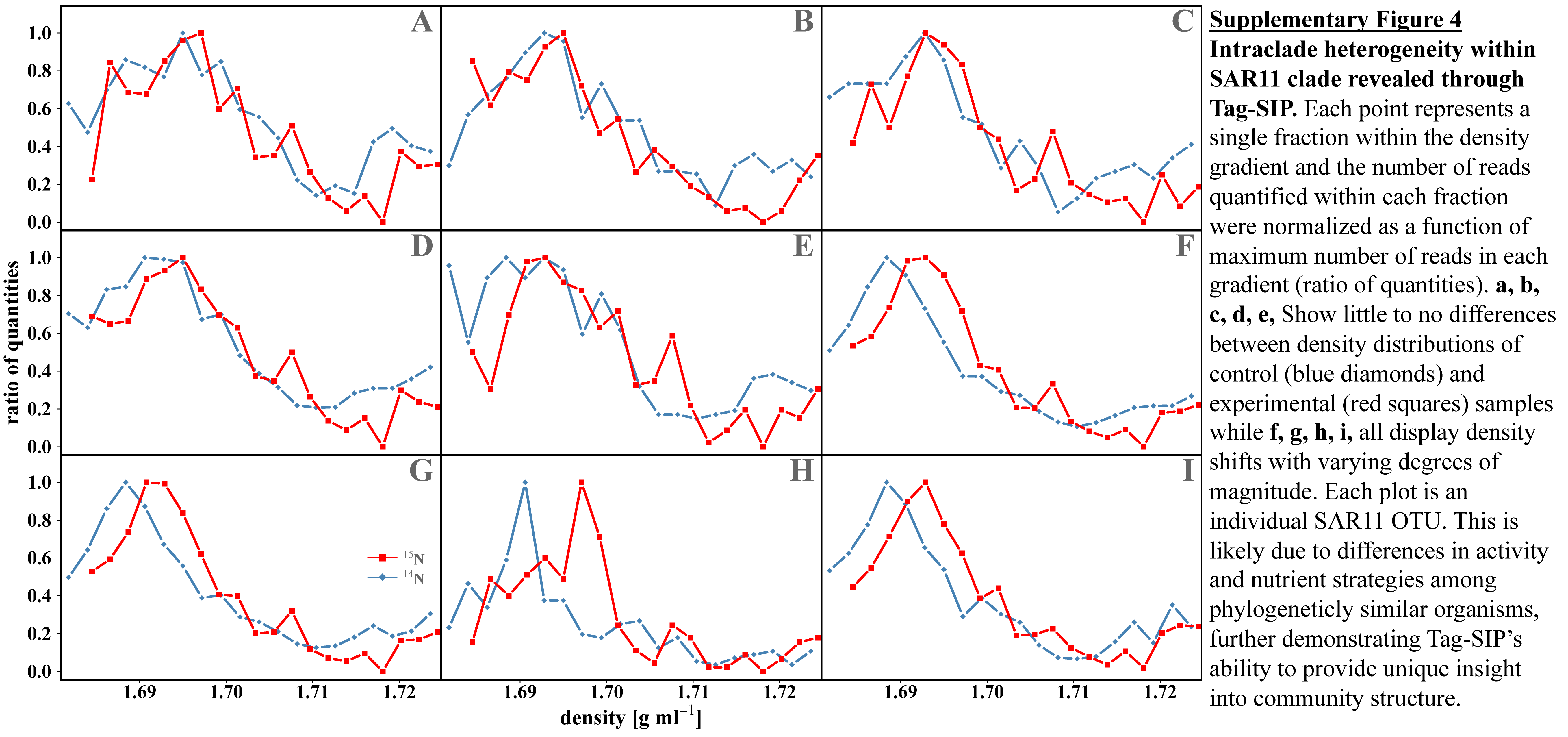

Supplement: Supplementary file 5 [file Image_4.TIF]

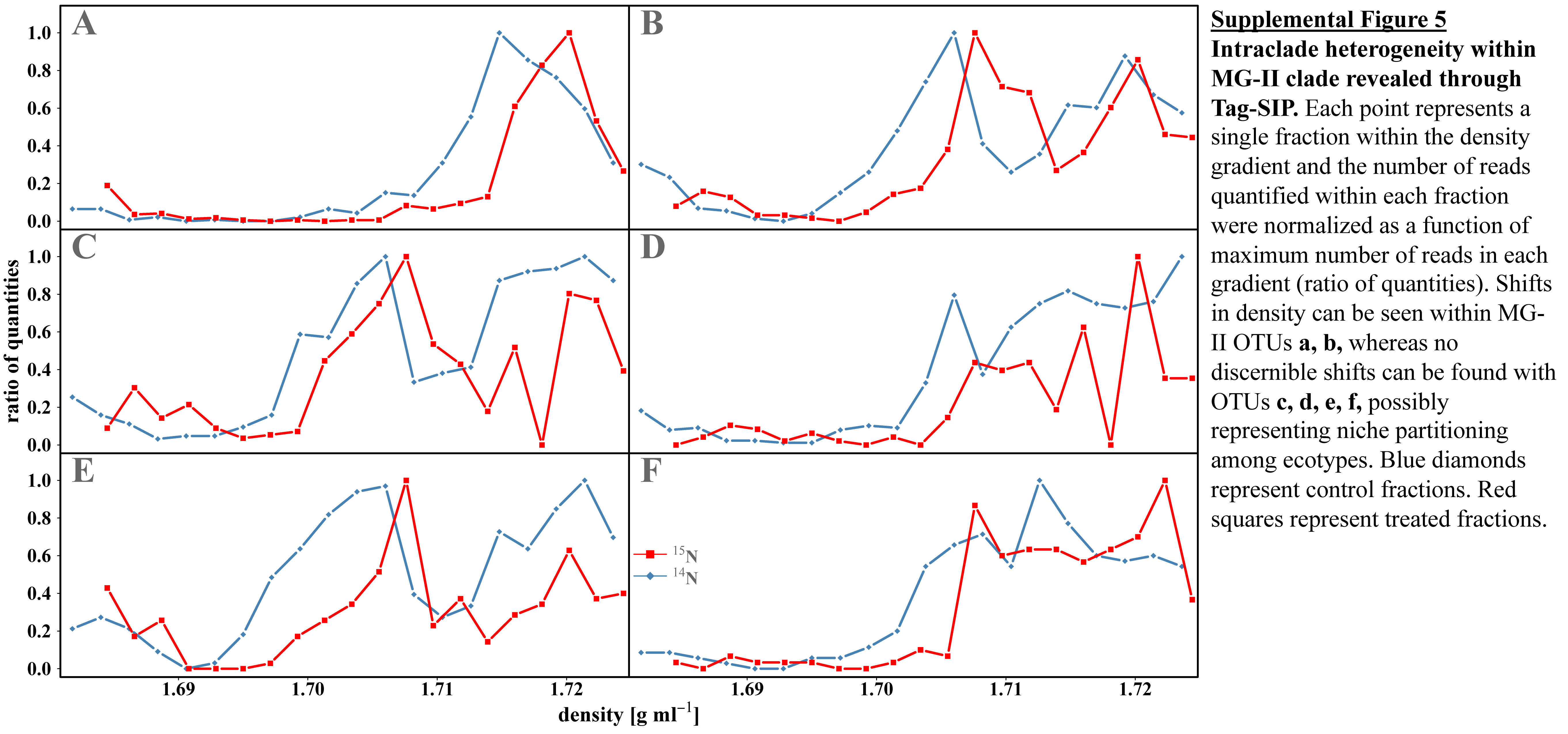

Supplement: Supplementary file 6 [file Image_5.TIF]

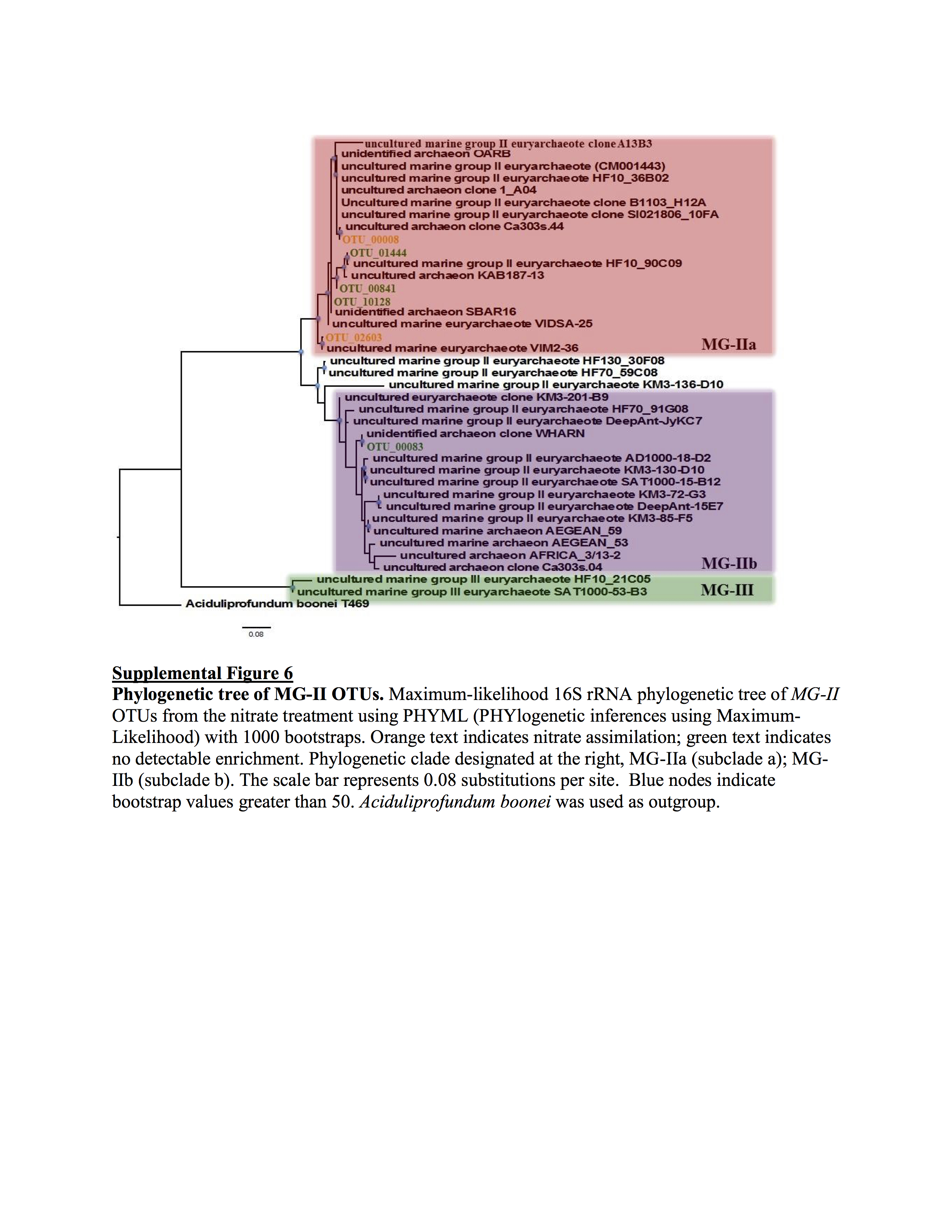

Supplement: Supplementary file 7 [file Image_6.JPEG]
